# Psychosocial Factors Associated with Quality of Life in Young Men Who Have Sex with Men Living with HIV/AIDS in Zhejiang, China

**DOI:** 10.3390/ijerph16152667

**Published:** 2019-07-25

**Authors:** Tingting Jiang, Xin Zhou, Hui Wang, Mingyu Luo, Xiaohong Pan, Qiaoqin Ma, Lin Chen

**Affiliations:** Zhejiang Provincial Center for Disease Control and Prevention, 3399 Binsheng Road, Hangzhou 310051, China

**Keywords:** young MSM, quality of life, social support, self-efficacy, stigma, antiviral therapy adherence

## Abstract

Objectives: To explore the quality of life (QOL) status and related factors in young human immunodeficiency virus (HIV)-infected men who have sex with men (MSM) aged 16 to 24 years in Zhejiang province. Methods: A cross-sectional study was conducted in 22 counties of Zhejiang province, and 395 subjects took part in our research. A *t*-test, one-way Analysis of variance (ANOVA), and multivariate stepwise linear regression analysis were used to investigate the factors associated with QOL in young HIV-infected MSM. Results: The total score on the QOL was 86.86 ± 14.01. The multivariate stepwise linear regression analysis revealed that self-efficacy and discrimination were associated with all domains on the QOL assessment, monthly income was associated with QOL for all domains except spirituality and consistent condom use during oral sex with men in the past three months was associated with QOL for all domains except the relationship domain. Those individuals within the group of young HIV-infected MSM who have higher self-efficacy, a higher monthly income, greater social support, safer sexual behaviors, a higher level of education, and a higher cluster of differentiation 4 (CD4) count have a better QOL. Conclusions: These findings suggest that to improve the QOL of this population, greater emphasis should be placed on improving social support, self-efficacy, and antiviral therapy adherence and on reducing discrimination, disease progression, and high-risk behaviors.

## 1. Introduction

Men who have sex with men (MSM) have become the group with the highest risk of human immunodeficiency virus (HIV) infection, exhibiting the fastest growth in HIV epidemic in China [1,2]. The MSM infection ratio in men aged 15 to 24 years old has increased progressively in recent years [3]. In 2017, more than 60 percent of the individuals newly infected with HIV were aged 15 to 24 years old among MSM in the Zhejiang province. Multiple sexual partners, high unprotected anal intercourse (UAI) behaviors, low condom use, and low HIV testing increased the risk of HIV infection in young MSM [4,5], and high risk behaviors put them at risk of HIV infection. The HIV infection problem in young MSM cannot be ignored in MSM HIV intervention.

Due to the application of a highly active antiretroviral therapy (HAART), the mortality rate from HIV/AIDS has significantly decreased and the life expectancy of HIV patients has increased, which means that HIV/AIDS is now considered a chronic, albeit controllable disease [6]. However, there are still many problems among HIV/AIDS patients with respect to the physical, psychological, cognitive, and social domains, especially among MSM with HIV/AIDS [7], who face not only the stress of having HIV but also the stigma and discrimination that are associated with having HIV and being part of the MSM community. 

Quality of life (QOL) can be defined as an individual’s satisfaction and subjective well-being with life in what she or he considers the most important domains, and it is interchangeable with “life satisfaction” [8,9]. Quality of life (QOL) is considered an important outcome measure in studies of HIV/AIDS treatment [10]. One UNAIDS survey [11] reflected a social trend of noting mental health when considering public health interventions to prevent and control HIV/AIDS, and it supported QOL as a ‘fourth 90’ for HIV/AIDS where 90% of people with viral load suppression have good health-related QOL. Learning more about QOL in MSM may help to understand their mental and physical status to improve their overall health [12].

Several studies [13,14] indicated that QOL is related to HIV-related behaviors, perceived discrimination [15], and self-efficacy, among others, etc. Furthermore, studies have found that self-efficacy is an important predictor of QOL among patients with chronic diseases [16,17]. A questionnaire survey [18] about China showed that self-efficacy, medication adherence, and drug use are important predictors of QOL. Another study found that HIV/AIDS patients having better engagement with health care providers have a better QOL, better self-efficacy for medication adherence, and reported fewer symptoms. All of the above indicated that self-efficacy is related to the QOL among people living with HIV/AIDS.

Social support has reported [19,20] to have a positive effect on the QOL of people living with HIV/AIDS. It is significant for people living with HIV/AIDS and their families to keep a positive attitude [21]. Studies [22,23] have revealed that social support is a protective factor on positive mental health among youths affected by HIV/AIDS. Social support can relieve stress and affect the patients’ physical well-being, mental health, and social function, thus affecting the QOL. Moreover, people living with HIV/AIDS with lower social support have a lower QOL [24,25].

Several studies have examined social support and QOL [12,14,26], as well as, self-efficacy and QOL [9,27] among people living with HIV/AIDS (PLWHA). However, there is limited research on self-efficacy, social support, and QOL among HIV-infected MSM in China, especially for young HIV-infected MSM aged 16 to 24 years. Accordingly, there is no evidence indicating which factors influence the QOL of young MSM with HIV/AIDS and the differences between these factors and those of other MSM patients with HIV/AIDS.

The aim of our study was to identify the factors that influence the QOL of young HIV-infected MSM in Zhejiang and determine the relationship between these factors and self-efficacy, social support, and other domains.

## 2. Materials and Methods

### 2.1. Study Design and Data Collection

A cross-sectional study was conducted in Zhejiang. The inclusion criteria were as follows: (1) Positive for HIV antibody; (2) infected through homosexual behavior; and (3) age ranging from 16 to 24 years old. First, we identified the counties that had more than 20 eligible patients in November 2016. There were 1583 eligible MSM patients; then, 400 participants were randomly selected from those 1583 patients. The exclusion criteria were the participants declining to participate. A replacement would be chosen via the same inclusion criteria in the local county. Thirteen replacements were ultimately recruited. Of 400 subjects, 5 participants were excluded from participating in the study due to a failure to provide key information, resulting in a total of 395 young MSM participants.

The sociodemographic and antiviral therapy information of the participants was collected from the case reporting database of the HIV/AIDS comprehensive response information management system in Zhejiang in November 2016, including age, level of education, marital status, employment, census register, infection status, route of transmission, and antiviral therapy status. We designed a questionnaire and collected the information including monthly income, self-identified sexual orientation, and route of dating, high-risk sexual behavior, and psychosocial and behavioral conditions such as self-efficacy, social support, and quality of life, etc.

### 2.2. Self-Efficacy

We used the HIV self-efficacy questionnaire (HIV-SE) to measure the self-efficacy of HIV disease management [28]. The questionnaire was revised by Chinese researchers based on feedback and cultural appropriateness. It contained 34 items divided among 6 domains: Emotional management (9 items), medication management (7 items), symptom management (5 items), communication with health care providers (4 items), receipt of support and assistance (5 items), and fatigue management (4 items). The participants responded to each item by indicating the confidence they felt in their abilities to perform each specific goal or behavior on a 10-point Likert scale ranging from 1 = “not at all sure” to 10 = “completely sure.” The instrument exhibited good reliability and construct validity. The Cronbach’s alpha coefficient was 0.94. Higher scores indicate that the respondent has better self-efficacy. Referring to Chen’s study [29], we divided the score for self-efficacy into three levels as follows: Scores ≥80% are high, between 79% and 41% are medium, and ≤40% are low.

### 2.3. Social Support

Social support was measured using the social support rating scale (SSRS) developed by the Chinese researcher Xiao [30]. The questionnaire included ten items in three dimensions: Objective support, subjective support, and support utilization, comprising three items, four items, and three items, respectively. Eight items had four possible responses, and the scores ranged from 1 to 4, two items had nine questions, participants could select more than one option, and each option was one score. The total score of questionnaire was the sum of the ten items; higher scores indicated greater social support. In this study, social support was divided into three support groups, namely, low, medium, and high, based on the total scores ranging from 25% to 75%. The Cronbach’s alpha coefficient of SSRS was 0.89.

### 2.4. Quality of Life

QOL was measured using the Chinese WHOQOL-HIV-BREF scale [31], which was translated from the WHOQOL-HIV-BREF for use in a Chinese population. This scale contained 31 items covering six dimensions and two additional general items (overall HRQOL and general health). The six domains are physical, psychological, independence, relationship, environment, and spirituality. The respondents were asked to rate certain experiences they had encountered over the past two weeks on a 5-point Likert scale ranging from 1 = very poor/dissatisfied to 5 = very good/satisfied. Higher scores indicated a better QOL. This tool has been determined to be valid and reliable for analysis of the MSM population. The internal consistency reliability ranged from 0.7 to 0.9 [16].

### 2.5. Statistical Analysis

Data were entered into EpiData version 3.1(EpiData Association, Odense, Denmark) and were processed using SPSS Statistics 19.0 (Armonk, NY, USA: IBM Corp) for statistical analysis. QOL was the continuous variable and it is presented as the mean ± standard deviation (SD). The QOL result was also compared with those from other studies using a *t*-test. A *t*-test and ANOVA were performed to investigate the relationships between and among demographic data, sexual behaviors, HIV infection, antiviral therapy, psychosocial factors, and QOL. A multivariate stepwise linear regression analysis was used to analyze the factors influencing QOL. The total QOL and domain scores were the dependent variable, and the variable *p* < 0.2 was incorporated into the independent variables. The method was stepwise, the entry variable was *p* < 0.05, and the removal variable was *p* > 0.10; 95%CIs and *p*-values were reported, when *p* < 0.05, the result was considered statistically significant.

### 2.6. Ethics Approval and Consent to Participate

This study was approved by the Ethics Committee of the Zhejiang Provincial Center for Disease Control and Prevention (Project Identification Code 2013-001) and informed consent was obtained before the survey was administered. We assured the respondents that the survey was confidential and that this survey would not have any negative impact on the respondents.

## 3. Results

### 3.1. QOL Score

The total QOL score was 86.86 ± 14.01, and the QOL scores by domain, namely, physical, psychological, independence, relationship, environment, and spirituality, were 15.19 ± 2.83, 14.28 ± 2.73, 15.62 ± 2.50, 13.58 ± 2.74, 13.93 ± 2.61, and 14.26 ± 3.35, respectively. The psychological, environment, and spirituality domains and total score for QOL were significantly higher than the national norm in China [32] (Table 1).

### 3.2. Sample Characteristics

Of the respondents, 96.7% (379/392) were single, 81.5% (322/395) were local residents, 52.9% (209/395) had a college education or higher, 56.0% (219/391) reported a monthly income ≥3000 RMB, 39.0% (154/395) were students when diagnosed, and 79.2% (309/390) self-reported their sexual orientation as homosexual (Table 2).

As shown in Table 3, 27.8% (110/395) were infected with a sexually transmitted disease (STD), 83.0% (328/395) had more than two male sexual partners in the past three months, only 20.5% (81/395) used condom consistently when having anal sex in the past three months, while 5.1% (20/395) used condom consistently when having oral sex in the past three months.

Table 4 shows that 88.9% (346/389) were on antiviral therapy, 26.6% (105/395) had AIDS, 61.8% (244/395) were infected for more than one year, 70.9% (280/395) were ≥350 on CD4 cell count, 20.5% (81/395) missed drugs (Table 4).

Psychosocial factors of the respondents, 20.1% (79/393) smoked cigarettes, 8.9% (35/393) used sexual aid, 66.1% (261/395) suffered discrimination, 38.7% (153/395) had a lower self-efficacy, and 27.1% (107/395) had a lower social support (Table 5).

### 3.3. Univariate Analysis of Factors and QOL

The results of the ANOVA and *t*-test are presented in Table 2, Table 3, Table 4 and Table 5. Of the 395 respondents, with respect to the total QOL score, MSM who had a higher level of education, who had a monthly income ≥3000 RMB, and who participated in online dating had a relatively higher total QOL score. Furthermore, monthly income, sexual orientation, and dating route were associated with QOL in the physical domain; the level of education, monthly income, employment status, and dating route were associated with QOL in the psychological, independence, and relationship domains; the level of education, monthly income, sexual orientation, employment status, and dating route were associated with QOL in the environment domain; and monthly income was associated with QOL in the spirituality domain (Table 2).

Table 3 indicates that consistent condom use during anal sex with men occurring within the past three months was associated with the total QOL score and with the scores for the independence and spirituality domains. Consistent condom use during oral sex with men occurring within the past three months was associated with all QOL domains except the relationship domain. Finally, young MSM who had numerous male sexual partners within the past three months had lower scores in the relationship domain.

With respect to disease, MSM who had AIDS exhibited lower scores in all QOL domains. MSM who had been infected for one or more years received higher QOL scores in the physical and spirituality domains, whereas those with CD4 counts ≥350 had higher total QOL scores as well as higher scores in the physical, psychological, and independence domains. Finally, taking drug on time is associated with a higher QOL score in the independence domain (Table 4).

The results of the relationships between QOL and psychosocial factors indicate that a lack of discrimination, higher self-efficacy, and greater social support are positively correlated with QOL. Young MSM who have higher self-efficacy and greater social support and who are not subjected to discrimination reported higher QOL scores in all domains (Table 5).

In a multivariate analysis (Table 6), the total QOL score was the dependent variable. The independent variables included the level of education, monthly income, sexual orientation, employment status, dating route, consistent condom use during anal and oral sex with men in the past three months, disease name, infection year, CD4 count, missing drugs, sexual aid use, discrimination, self-efficacy, and social support. The results revealed that self-efficacy (β = 8.071, *p* < 0.001), discrimination (β = −8.338, *p* = 0.000), monthly income (β = 5.398, *p* < 0.001), employment status (β = −1.344, *p* = 0.022), condom use during oral sex with men in the past three months (β = 2.131, *p* = 0.001), social support (β = 1.692, *p* = 0.016), and disease progression (β = −2.491, *p* = 0.032) are the factors associated with QOL. More specifically, the results indicated that a higher self-efficacy, monthly income ≥3000 RMB, student status, condom use during oral sex with men in the past three months, and greater social support are associated with better QOL values. In addition, discrimination and AIDS were negatively related to QOL. For example, when young MSM experienced discrimination at the AIDS stage, they demonstrated a lower QOL. With respect to psychosocial factors, self-efficacy and discrimination were associated with QOL in all domains. Additionally, social support was associated with the relationship and spirituality domains, while smoking was related to the physical domain. Regarding sociodemographic factors, monthly income was associated with QOL in all domains except spirituality; employment status was associated with the psychological, independence and relationship domains; sexual orientation was associated with the physical and environment domains; and level of education and dating route were associated with the environment domain. With respect to sexual factors, the number of male sexual partners within the past three months was associated with the relationship domain, and condom use during oral sex with men occurring within the past three months was associated with QOL in all domains except the relationship domain. With respect to antiviral therapy factors, the CD4 count was associated with the physical and independence domains, disease progression was associated with the psychological domain and missing drugs was associated with the independence domain.

## 4. Discussion

The aim of this study was to assess the QOL of young HIV-infected MSM and the relationship between QOL and various influencing factors, such as social support and self-efficacy, in Zhejiang and in the eastern coastal cities of China. As a measurement of physical, psychological, and social adaption indicators, QOL reflects the health status of people living with HIV/AIDS (PLWHA) [33]. In this study, the QOL for psychological, environment, and spirituality domains and total score were significantly higher than in the general population in China. Furthermore, the QOL scores were significantly higher than other populations of PLWHA [34,35]. Ma et al.’s study (2015) [36] on PLWHA in Zhejiang also stated that younger PLWHA demonstrated higher QOL scores. This finding implies that the QOL of young HIV-infected MSM in Zhejiang is better than those of other populations of PLWHA.

In our study, the results indicated that self-efficacy and discrimination were significantly correlated with all QOL domains. Specifically, increased self-efficacy resulted in higher QOL scores among young MSM. The study of Cramm et al. (2013) [37] demonstrated that general self-efficacy was an important factor that influences the social QOL in youths with chronic conditions. Chen et al.’s study (2013) [9] concluded that health care providers improved QOL among PLWHA by enhancing their self-efficacy and self-esteem. Specifically, the results indicated that self-efficacy was a significant predictive factor of QOL in the population of young HIV-infected MSM. Our findings further revealed that stigma and discrimination were negatively associated with QOL in all domains. Although MSM anti-discrimination propaganda has been promoted in China, stigma and discrimination related to sexual orientation still exist. Thus, MSM are inclined to hide their homosexual identities. Moreover, such shame and discrimination are major barriers that cause MSM and young HIV-infected MSM to hesitate to seek health services [38,39]. In addition, HIV-related stigma and fear evoke negative emotions among most HIV-infected MSM [40]. Both are factors that contribute to reducing the QOL of this population.

Our study showed that social support was positively associated with the total QOL scores and with the scores for the relationship and spirituality domains. Social support helps young MSM cope with stressful life events and adapt to life. Peter et al.’s study from southern India [41] revealed that health-related QOL was positively associated with social support from family and friends. Lan et al.’s study (2015) [33] concluded that subjective support and the use of social support were positively correlated with QOL. These findings indicate that there should be a focus on improving the social support provided to young HIV-infected MSM because increased social support can help individuals more effectively cope with their difficult situation [42]. Moreover, social support plays an important role in reducing risky sexual behaviors [43], whereas a lack of social support may result in increased risky sexual behaviors; accordingly, reducing risky sexual behaviors by improving social support can result in an improved QOL.

In our study, young MSM reported a high number of sexual partners; specifically, the prevalence of young HIV-infected MSM who had two or more male sexual partners within the past three months was 83%. Moreover, the use of condoms during oral sex occurring within the past three months was positively associated with the total QOL score and with all domains except the relationship domain. Young MSM who had more male sexual partners had lower QOL scores in the relationship domain. Young MSM who smoked exhibited an impaired score in the physical domain of the QOL. It was also determined that the young MSM population engaged in high-risk sexual behaviors [44,45,46] and that there was a relationship between high-risk behavior and QOL. For example, high-risk behaviors were associated with negative emotions, such as depression and anxiety, and higher depression and anxiety scores were associated with lower QOL scores in the physical, psychological, and relationship domains [41,47].

Among the sociodemographic factors, monthly income was found to be related to QOL, a finding that is consistent with those of previous studies [14,48]. In this study, increased monthly income was positively related to QOL, while higher income was correlated with a better QOL in all domains except spirituality. In the present study, the level of education and dating route influenced the environmental domain. Specifically, those with a higher level of education and those who made friends at school exhibited higher QOL scores. Similarly, young MSM who accepted their homosexuality reported better QOL scores in the physical and environmental domains. The higher QOL scores among these populations may be because young MSM are more open-minded and more accepting of their homosexuality, especially within higher education populations. Both of these groups demonstrated more enlightened attitudes toward HIV.

Although no correlation was found between antiviral therapy and QOL in our study, the CD4 count was correlated with better scores in the physical and independence domains. Nachega et al.’s study (2011) [49] indicated that interrupted ART limited the recovery of CD4 and increased the risk of opportunistic complications and death. Additionally, missing drugs was also an important factor correlated with medication adherence, with missing drugs exhibiting lower scores in the independence domain. Furthermore, medication adherence is positively correlated with QOL, such that people with a high QOL have a higher adherence to treatment, while low adherence to ART may result in an adverse effect, causing people to exhibit poorer health, more physical symptoms, and a lower QOL [50].

We also found a negative association between young MSM who were in the AIDS stage and the total QOL score, particularly in the score for the psychological domain. This result may be because AIDS patients experience more symptoms and increased complications as well as increased psychosocial problems, which together may result in poorer QOL for AIDS patients.

This study has several limitations. First, as this study was based on a cross-sectional survey, it is difficult to describe variation tendencies with respect to self-efficacy, social support, and QOL. Furthermore, causal relationships between QOL and related factors cannot be determined. Thus, a future investigation should conduct a cohort study on QOL. Second, to facilitate the investigation, randomly selected samples were obtained from counties where there were more than 20 cases of young HIV-infected MSM. This, however, is not a truly random sampling; therefore, the sampling method should be improved in a future study. Third, stigma and discrimination are acknowledged as important factors with respect to the QOL of PLWHA, as PLWHA are subjected to more negative stigma and greater discrimination than other groups. In our study, we used only one question to measure stigma and discrimination. Thus, it is recommended that future studies use appropriate scales to measure stigma and discrimination and to better measure the association between discrimination and QOL.

## 5. Conclusions

Our study revealed that there is a higher QOL among young HIV-infected MSM than among other HIV/AIDS patients. Factors such as social support, self-efficacy, income level, and high CD4 count have a protective effect on the QOL in young HIV-infected MSM, whereas discrimination, AIDS stage, and high-risk behaviors have a negative effect on QOL. The results imply that we should focus on and enhance psychosocial factors, such as social support, self-efficacy, and anti-discrimination, and strengthen the strategies and techniques to diagnose HIV and discover and treat young HIV-infected individuals earlier. Such changes will help to control the disease in its HIV stage through treatment, reduce the complications associated with the disease, and improve the QOL of infected individuals. Finally, agencies and involved individuals must advocate for safe sex behaviors to reduce the possibilities of coinfection.

## Figures and Tables

**Table 1:** Quality of life scores compared with national norm in China.

Quality of Life	Mean Scores	National Norm	*t*	*p*
Physical	15.19 ± 2.83	15.10 ± 2.30	0.58	>0.05
Psychological	14.28 ± 2.73	13.89 ± 1.89	2.85	<0.05
Independence	15.62 ± 2.50	15.64 ± 2.22	−0.14	>0.05
Relationship	13.58 ± 2.74	13.93 ± 2.06	−2.45	>0.05
Environment	13.93 ± 2.61	12.14 ± 2.08	12.75	<0.05
Spirituality	14.26 ± 3.35	11.05 ± 3.68	11.46	<0.05
Total score	86.86 ± 14.01	80.28 ± 17.46	6.50	<0.05

**Table 2:** Sociodemographic characteristics and relationships with all domains of quality of life for all participants.

Variables	Number	Physical	Psychological	Independence	Relationship	Environment	Spirituality	Total Score
Area Source								
inner province	322	15.14 ± 2.88	14.25 ± 2.78	15.61 ± 2.48	13.56 ± 2.80	13.96 ± 2.61	14.25 ± 3.34	86.76 ± 14.18
Other province	73	15.42 ± 2.61	14.41 ± 2.49	15.65 ± 2.60	13.68 ± 2.49	13.82 ± 2.64	14.32 ± 3.41	87.32 ± 13.33
	T	−0.784	−0.452	−0.160	−0.363	0.397	−0.146	−0.307
	*p*	0.434	0.651	0.873	0.717	0.691	0.392	0.759
Age (22.02 ± 1.789)								
16 to 19	41	14.61 ± 3.39	13.89 ± 3.21	15.15 ± 2.57	13.49 ± 3.07	13.51 ± 2.80	13.66 ± 2.91	84.31 ± 15.60
20 and older	354	15.26 ± 2.76	14.32 ± 2.67	15.67 ± 2.49	13.59 ± 2.70	13.98 ± 2.59	14.33 ± 3.39	87.16 ± 13.81
	T	−1.386	−0.962	−1.268	−0.227	−1.087	−1.223	−1.233
	*p*	0.167	0.337	0.206	0.821	0.278	0.222	0.218
Marital Status								
Single	379	15.18 ± 2.81	14.28 ± 2.73	15.59 ± 2.48	13.57 ± 2.75	13.96 ± 2.58	14.25 ± 3.32	86.83 ± 13.89
Other	13	15.07 ± 3.77	14.34 ± 2.97	16.23 ± 3.44	13.54 ± 2.85	13.08 ± 3.53	14.54 ± 4.27	86.80 ± 18.79
	T	0.131	−0.082	−0.906	0.044	1.193	−0.304	0.007
	*p*	0.896	0.935	0.365	0.965	0.234	0.761	0.995
Educational Level								
Junior high or lower	76	14.66 ± 3.16	13.45 ± 2.63	14.91 ± 2.65	12.58 ± 2.41	12.58 ± 2.44	13.83 ± 2.84	82.01 ± 13.17
High school	110	15.11 ± 2.82	14.16 ± 2.84	15.56 ± 2.56	13.57 ± 2.67	14.02 ± 2.60	14.10 ± 3.35	86.51 ± 14.24
College or higher	209	15.43 ± 2.70	14.65 ± 2.64	15.90 ± 2.37	13.95 ± 2.81	14.38 ± 2.52	14.51 ± 3.50	88.81 ± 13.81
	F	2.120	5.607	4.482	7.173	14.217	1.365	6.807
	*p*	0.121	0.004	0.012	0.001	<0.001	0.257	0.001
Monthly Income (RMB)								
<3000	172	14.52 ± 3.01	13.60 ± 3.03	14.89 ± 2.69	13.08 ± 2.88	13.22 ± 2.82	13.76 ± 3.45	83.08 ± 15.17
≥3000	219	15.70 ± 2.59	14.79 ± 2.36	16.16 ± 2.21	13.94 ± 2.58	14.47 ± 2.31	14.57 ± 3.20	89.63 ± 12.35
	T	−4.167	−4.339	−5.127	−3.106	−4.794	−2.411	−4.707
	*p*	<0.001	<0.001	<0.001	0.002	<0.001	0.016	<0.001
Sexual orientation								
Homosexual	309	15.37 ± 2.77	14.34 ± 2.75	15.63 ± 2.54	13.64 ± 2.78	14.09 ± 2.60	14.20 ± 3.28	87.28 ± 13.87
Other	81	14.44 ± 2.96	14.04 ± 2.68	15.46 ± 2.42	13.35 ± 2.64	13.26 ± 2.61	14.44 ± 3.66	85.03 ± 14.75
	T	2.662	0.851	0.526	0.814	2.560	−0.573	1.288
	*p*	0.008	0.396	0.599	0.416	0.011	0.567	0.198
Employment status								
Student	154	15.41 ± 2.84	14.68 ± 2.67	15.89 ± 2.37	14.25 ± 2.63	14.37 ± 2.45	14.64 ± 3.24	89.25 ± 13.56
Commercial service	68	15.01 ± 2.67	14.47 ± 2.23	15.93 ± 2.20	13.65 ± 2.35	14.04 ± 2.21	14.22 ± 3.21	87.32 ± 10.94
Other	173	15.06 ± 2.89	13.85 ± 2.90	15.24 ± 2.69	12.96 ± 2.84	13.49 ± 2.83	13.94 ± 3.48	84.75 ± 15.09
	F	0.719	4.044	3.452	9.406	4.788	1.863	4.707
	*p*	0.488	0.018	0.033	<0.001	0.009	0.157	0.010
Dating route								
Traditional mode	53	14.32 ± 2.32	13.33 ± 2.50	14.81 ± 1.86	12.66 ± 2.49	12.58 ± 2.48	13.60 ± 3.06	81.30 ± 12.13
Internet	281	15.22 ± 2.95	14.28 ± 2.76	15.61 ± 2.52	13.55 ± 2.74	14.01 ± 2.53	14.20 ± 3.39	86.86 ± 14.11
Other (mainly at school)	54	15.81 ± 2.64	15.17 ± 2.62	16.30 ± 2.89	14.72 ± 2.68	14.82 ± 2.85	15.13 ± 3.38	91.96 ± 14.09
	F	3.821	6.186	4.758	7.913	10.794	2.888	7.916
	*p*	0.023	0.002	0.009	<0.001	<0.001	0.057	<0.001

**Table 3:** Sexual factors and their associations with quality of life (*n* = 395).

Variables	Number	Physical	Psychological	Independence	Relationship	Environment	Spirituality	Total Score
STD History								
Yes	110	15.03 ± 2.79	13.95 ± 2.68	15.58 ± 2.59	13.44 ± 2.68	13.70 ± 2.52	13.95 ± 2.91	85.64 ± 12.99
No	285	15.25 ± 2.85	14.41 ± 2.74	15.63 ± 2.47	13.64 ± 2.77	14.02 ± 2.64	14.39 ± 3.50	87.33 ± 14.38
	T	−0.708	−1.503	−0.164	−0.646	−1.118	−1.173	−1.079
	*p*	0.479	0.134	0.870	0.519	0.264	0.242	0.281
Number of Male Sexual Partners in the Past 3 Months								
0–1	67	15.34 ± 2.99	14.61 ± 2.45	15.58 ± 2.30	14.13 ± 2.67	14.10 ± 2.42	14.15 ± 3.22	87.93 ± 13.59
2–9	233	15.17 ± 2.79	14.29 ± 2.75	15.66 ± 2.62	13.63 ± 2.74	14.03 ± 2.64	14.44 ± 3.37	87.22 ± 14.24
10 or more	95	15.14 ± 2.86	14.03 ± 2.86	15.62 ± 2.50	13.06 ± 2.72	13.57 ± 2.65	13.90 ± 3.39	85.23 ± 13.77
	F	0.122	0.907	0.104	3.137	1.200	0.914	0.908
	*p*	0.886	0.405	0.901	0.045	0.302	0.402	0.404
Consistent Condom Use during Anal Sex with Men in the Past 3 Months								
No	42	14.38 ± 2.60	13.58 ± 2.45	14.38 ± 2.70	12.90 ± 2.99	13.08 ± 3.04	13.00 ± 3.50	81.33 ± 13.51
Yes	81	14.93 ± 2.88	14.02 ± 2.66	15.70 ± 2.41	13.62 ± 2.68	13.78 ± 2.53	13.91 ± 3.37	85.97 ± 13.07
Never occurred	272	15.39 ± 2.84	14.46 ± 2.77	15.78 ± 2.46	13.67 ± 2.71	14.11 ± 2.54	14.56 ± 3.27	87.98 ± 14.19
	F	2.788	2.376	5.882	1.443	2.989	4.602	4.374
	*p*	0.063	0.094	0.003	0.237	0.051	0.011	0.013
Consistent Condom Use during Oral Sex with Men in the Past 3 Months								
No	74	14.03 ± 2.86	13.24 ± 2.72	14.80 ± 2.67	13.07 ± 2.83	13.12 ± 2.88	13.30 ± 3.23	81.55 ± 13.73
Yes	20	14.85 ± 2.58	14.36 ± 2.04	15.75 ± 2.63	14.10 ± 2.10	13.95 ± 1.83	13.25 ± 3.42	86.26 ± 10.03
Never occurred	301	15.50 ± 2.77	14.53 ± 2.71	15.81 ± 2.42	13.67 ± 2.74	14.13 ± 2.55	14.57 ± 3.32	88.20 ± 14.03
	F	8.464	6.822	4.962	1.829	4.508	5.362	6.909
	*p*	<0.001	0.001	0.007	0.162	0.012	0.005	0.001

STD: sexually transmitted disease.

**Table 4:** HIV infection, antiviral therapy factors, and their relationships with quality of life (*n* = 395).

Variables	Number	Physical	Psychological	Independence	Relationship	Environment	Spirituality	Total Score
Disease Progression								
HIV	290	15.38 ± 2.86	14.56 ± 2.68	15.86 ± 2.48	13.80 ± 2.68	14.15 ± 2.63	14.48 ± 3.26	88.24 ± 13.77
AIDS	105	14.66 ± 2.70	13.50 ± 2.71	14.93 ± 2.47	12.97 ± 2.82	13.32 ± 2.48	13.67 ± 3.51	83.05 ± 14.04
	T	2.260	3.467	3.298	2.677	2.832	2.141	3.293
	*p*	0.024	0.001	0.001	0.008	0.005	0.033	0.001
Infection Years								
<1 year	151	14.76 ± 2.88	14.06 ± 2.88	15.32 ± 2.57	13.28 ± 2.86	13.65 ± 2.66	13.83 ± 3.45	84.90 ± 14.65
≥1 year	244	15.46 ± 2.78	14.42 ± 2.62	15.80 ± 2.45	13.77 ± 2.65	14.10 ± 2.57	14.53 ± 3.26	88.07 ± 13.49
	T	−2.414	−1.291	−1.821	−1.726	−1.677	−2.011	−1.955
	*p*	0.016	0.198	0.069	0.085	0.094	0.045	0.029
CD4 Count								
<350	115	14.66 ± 2.93	13.88 ± 2.81	15.06 ± 2.54	13.29 ± 2.72	13.66 ± 2.66	13.88 ± 3.51	84.42 ± 14.50
≥350	280	15.41 ± 2.77	14.45 ± 2.68	15.84 ± 2.46	13.70 ± 2.74	14.04 ± 2.59	14.42 ± 3.27	87.86 ± 13.71
	T	−2.392	−1.885	−2.845	−1.363	−1.344	−1.467	−2.227
	*p*	0.017	0.06	0.005	0.174	0.180	0.143	0.026
Antiviral Therapy								
No	43	15.74 ± 2.88	14.62 ± 2.95	16.00 ± 2.42	13.88 ± 2.80	14.33 ± 2.78	14.49 ± 2.84	86.75 ± 13.93
Yes	346	15.13 ± 2.83	14.26 ± 2.69	15.60 ± 2.48	13.58 ± 2.70	13.93 ± 2.57	14.25 ± 3.40	89.07 ± 13.94
	T	1.341	0.824	0.997	0.684	0.943	0.449	1.027
	*p*	0.181	0.410	0.319	0.494	0.346	0.653	0.305
Missing Drugs								
Yes	81	14.67 ± 2.88	13.96 ± 2.51	14.96 ± 2.30	13.41 ± 2.58	13.72 ± 2.52	13.85 ± 3.45	84.56 ± 13.44
No	314	15.32 ± 2.81	14.36 ± 2.78	15.78 ± 2.53	13.62 ± 2.78	13.99 ± 2.63	14.37 ± 3.32	87.45 ± 14.12
	T	−1.869	−1.204	−2.650	−0.635	−0.833	−1.242	−1.660
	*p*	0.062	0.229	0.008	0.526	0.405	0.215	0.098

**Table 5:** Psychosocial factors and their associations with quality of life (*n* = 395).

Variables	Number	Physical	Psychological	Independence	Relationship	Environment	Spirituality	Total Score
Smoking								
Yes	79	14.70 ± 3.09	14.13 ± 2.88	15.54 ± 2.61	13.84 ± 2.98	13.99 ± 2.94	14.48 ± 3.51	86.67 ± 15.128
No	314	15.32 ± 2.76	14.32 ± 2.70	15.62 ± 2.49	13.51 ± 2.68	13.91 ± 2.53	14.20 ± 3.31	86.88 ± 13.785
	T	−1.740	−0.572	−0.253	0.943	0.227	0.679	−0.118
	*p*	0.083	0.567	0.801	0.346	0.820	0.497	0.906
Sexual Aid Use								
Yes	35	14.46 ± 2.72	13.44 ± 2.67	15.34 ± 2.18	13.29 ± 2.64	13.64 ± 2.51	13.51 ± 2.55	83.68 ± 11.102
No	358	15.26 ± 2.84	14.37 ± 2.73	15.63 ± 2.54	13.60 ± 2.76	13.96 ± 2.63	14.32 ± 3.41	87.15 ± 14.276
	T	−1.608	−1.921	−0.655	−0.653	−0.674	−1.366	−1.394
	*p*	0.109	0.055	0.513	0.514	0.501	0.173	0.164
Self-Efficacy								
Low	153	13.87 ± 2.74	12.67 ± 2.68	14.33 ± 2.47	12.05 ± 2.45	12.42 ± 2.35	12.88 ± 3.09	78.22 ± 13.090
Middle	163	15.40 ± 2.53	14.73 ± 2.05	15.97 ± 2.04	13.98 ± 2.07	14.28 ± 2.11	14.60 ± 3.02	88.97 ± 10.471
High	79	17.30 ± 2.16	16.47 ± 2.13	17.37 ± 2.13	15.71 ± 2.80	16.15 ± 2.16	16.25 ± 3.31	99.24 ± 10.918
	F	48.463	74.669	51.557	65.610	77.297	32.383	89.609
	*p*	<0.001	<0.001	<0.001	<0.001	<0.001	<0.001	<0.001
Discrimination								
No	134	16.67 ± 2.57	15.46 ± 2.36	16.72 ± 2.06	14.90 ± 2.52	14.94 ± 2.33	16.74 ± 2.18	95.44 ± 10.957
Yes	261	14.43 ± 2.66	13.67 ± 2.71	15.05 ± 2.52	12.90 ± 2.60	13.42 ± 2.60	12.99 ± 3.13	82.46 ± 13.373
	T	8.022	6.489	6.643	7.325	5.698	12.409	9.687
	*p*	<0.001	<0.001	<0.001	<0.001	<0.001	<0.001	<0.001
Social Support								
Low	107	14.53 ± 3.20	13.62 ± 3.06	14.81 ± 2.67	12.64 ± 2.84	13.12 ± 2.71	13.22 ± 3.44	81.93 ± 14.92
middle	177	15.41 ± 2.55	14.44 ± 2.54	16.02 ± 2.21	13.75 ± 2.35	14.11 ± 2.43	14.44 ± 3.18	88.18 ± 12.45
High	111	15.47 ± 2.82	14.66 ± 2.58	15.74 ± 2.62	14.22 ± 3.00	14.42 ± 2.63	15.00 ± 3.31	89.51 ± 14.01
	F	4.019	4.693	8.264	10.152	7.813	8.479	9.802
	*p*	0.019	0.010	<0.001	<0.001	<0.001	<0.001	<0.001

**Table 6:** Multiple regression analysis of factors associated with quality of life (*n* = 395).

Domains	Variable	B	SE	*t*	*p*	B: 95% CI	
						Lower Bound	Upper Bound
Physical	Self-efficacy	1.348	0.164	8.235	<0.001	1.026	1.670
	Discrimination	−1.690	0.258	−6.549	<0.001	−2.197	−1.182
	Monthly income	1.136	0.238	4.776	<0.001	0.669	1.604
	Consistent condom use during oral sex with men	0.512	0.150	3.416	0.001	0.217	0.807
	Sexual orientation	−0.999	0.293	−3.411	0.001	−1.575	−0.423
	Smoking or not	0.935	0.298	3.137	0.002	0.349	1.521
	CD4 count	0.562	0.259	2.167	0.031	0.052	1.072
Psychological	Self-efficacy	1.649	0.158	10.405	<0.001	1.337	1.961
	Monthly income	1.158	0.232	4.993	<0.001	0.702	1.614
	Discrimination	−1.006	0.244	−4.120	<0.001	−1.486	−0.526
	Consistent condom use during oral sex with men	0.459	0.143	3.213	0.001	0.178	0.740
	Disease progression	−0.624	0.255	−2.445	0.015	−1.125	−0.122
	Employment status	−0.298	0.128	−2.334	0.020	−0.549	−0.047
Independence	Self-efficacy	1.257	0.149	8.453	<0.001	0.964	1.549
	Monthly income	1.307	0.217	6.026	<0.001	0.880	1.733
	Discrimination	−1.074	0.228	−4.704	<0.001	−1.522	−0.625
	CD4 count	0.537	0.232	2.314	0.021	0.081	0.993
	Consistent condom use during oral sex with men	0.318	0.133	2.383	0.018	0.056	0.581
	Employment status	−0.288	0.120	−2.398	0.017	−0.523	−0.052
	Missing drugs	−0.602	0.265	−2.275	0.023	−1.123	−0.082
Relationship	Self-efficacy	1.503	0.160	9.380	<0.001	1.188	1.818
	Discrimination	−1.291	0.246	−5.237	<0.001	−1.775	−0.806
	Monthly income	0.960	0.234	4.097	<0.001	0.499	1.420
	Employment status	−0.437	0.129	−3.380	0.001	−0.691	−0.183
	Social support	0.421	0.154	2.742	0.006	0.119	0.723
	Number of male sexual partners in the past 6 months	−0.378	0.179	−2.109	0.036	−0.730	−0.026
Environment	Self-efficacy	1.578	0.149	10.607	<0.001	1.285	1.870
	Monthly income	1.086	0.210	5.177	<0.001	0.674	1.499
	Educational level	0.536	0.134	3.992	<0.001	0.272	0.800
	Discrimination	−0.714	0.227	−3.142	0.002	−1.161	−0.267
	Sexual orientation	−0.952	0.260	−3.655	0.000	−1.464	−0.440
	Dating route	0.624	0.206	3.034	0.003	0.220	1.029
	Consistent condom use during oral sex with men	0.361	0.133	2.724	0.007	0.100	0.622
Spirituality	Discrimination	−3.189	0.300	−10.631	<0.001	−3.779	−2.599
	Self-efficacy	1.062	0.191	5.545	<0.001	0.685	1.438
	Consistent condom use during oral sex with men	0.586	0.175	3.353	0.001	0.243	0.930
	Social support	0.588	0.185	3.172	0.002	0.224	0.953
Total QOL Score	Self-efficacy	8.369	0.746	11.218	<0.001	6.902	9.836
	Discrimination	−8.655	1.145	−7.556	<0.001	−10.907	−6.402
	Monthly income	6.022	1.100	5.475	<0.001	3.860	8.185
	Consistent condom use during oral sex with men in the past 3 months	2.488	0.670	3.711	<0.001	1.170	3.806
	Employment status	−1.534	0.602	−2.548	0.011	−2.717	−0.350
	Social support	1.772	0.719	2.463	0.014	0.357	3.186
	Disease progression	−2.784	1.193	−2.334	0.020	−5.130	−0.438

## Abbreviations

QOL	quality of life
MSM	men who have sex with men
WHOQOL-HIV-BREF	the World Health Organization Quality of life Questionnaire for HIV Brief Version
HAART	highly active antiretroviral therapy
ANOVA	Analysis of variance
CD4	cluster of differentiation 4
UAI	unprotected anal intercourse
SD	standard deviation
HIV-SE	HIV self-efficacy questionnaire
SSRS	Social support rating scale
PLWHA	people living with HIV/AIDS
STD	sexually transmitted disease
